# Myocardial T2 signal enhancement in hypertrophic cardiomyopathy: prevalence, clinical profile and pathologic correlation

**DOI:** 10.1186/1532-429X-16-S1-O85

**Published:** 2014-01-16

**Authors:** Louis Kolman, John Stirrat, Martin Rajchl, Sebastien X Joncas, Yoko Mikami, Edward J Tweedie, Jacqueline Flewitt, Carmen Lydell, Andrew G Howarth, James A White

**Affiliations:** 1 Stephenson CMR Center, University of Calgary, Calgary, Alberta, Canada; 2 Western University, London, Ontario, Canada

## Background

Abnormal myocardial T2 signal has been described in a minority of patients with hypertrophic cardiomyopathy (HCM). This phenomenon has been associated with arrhythmic outcomes and, given its more restrictive prevalence versus late gadolinium enhancement (LGE), offers a potentially useful biomarker for risk stratification. To date, postulated mechanisms include active tissue disease or unique collagen species, however, no histopathologic correlation has been reported. In this study, we examine the prevalence of T2 signal abnormalities among patients with HCM and examine for associations with other imaging-based markers of HCM phenotypic expression. Incrementally, we provide comprehensive histopathologic correlation of a patient undergoing cardiac transplantation receiving detailed in-vivo and ex-vivo (explanted heart) imaging.

## Methods

Consecutive patients with HCM underwent a standardized imaging protocol using a 3-Tesla MR system. This was inclusive of T2-weighted imaging using a short-Tau inversion-recovery (STIR) fast spin echo sequence in serial short axis imaging planes both with and without body surface coils. Cine and LGE imaging was performed in identical slice orientations. Blinded analysis of left ventricular (LV) wall thickness, LV volumes, T2 signal enhancement and LGE signal abnormalities were performed using commercial software. In a patient planned for transplantation, a detailed in-vivo and ex-vivo imaging protocol was performed, the latter performing high-resolution isotropic T2-weighted imaging. Tissue was sampled from regions with and without abnormal T2 signal and exposed to comprehensive histologic examination.

## Results

**Figure 1:**
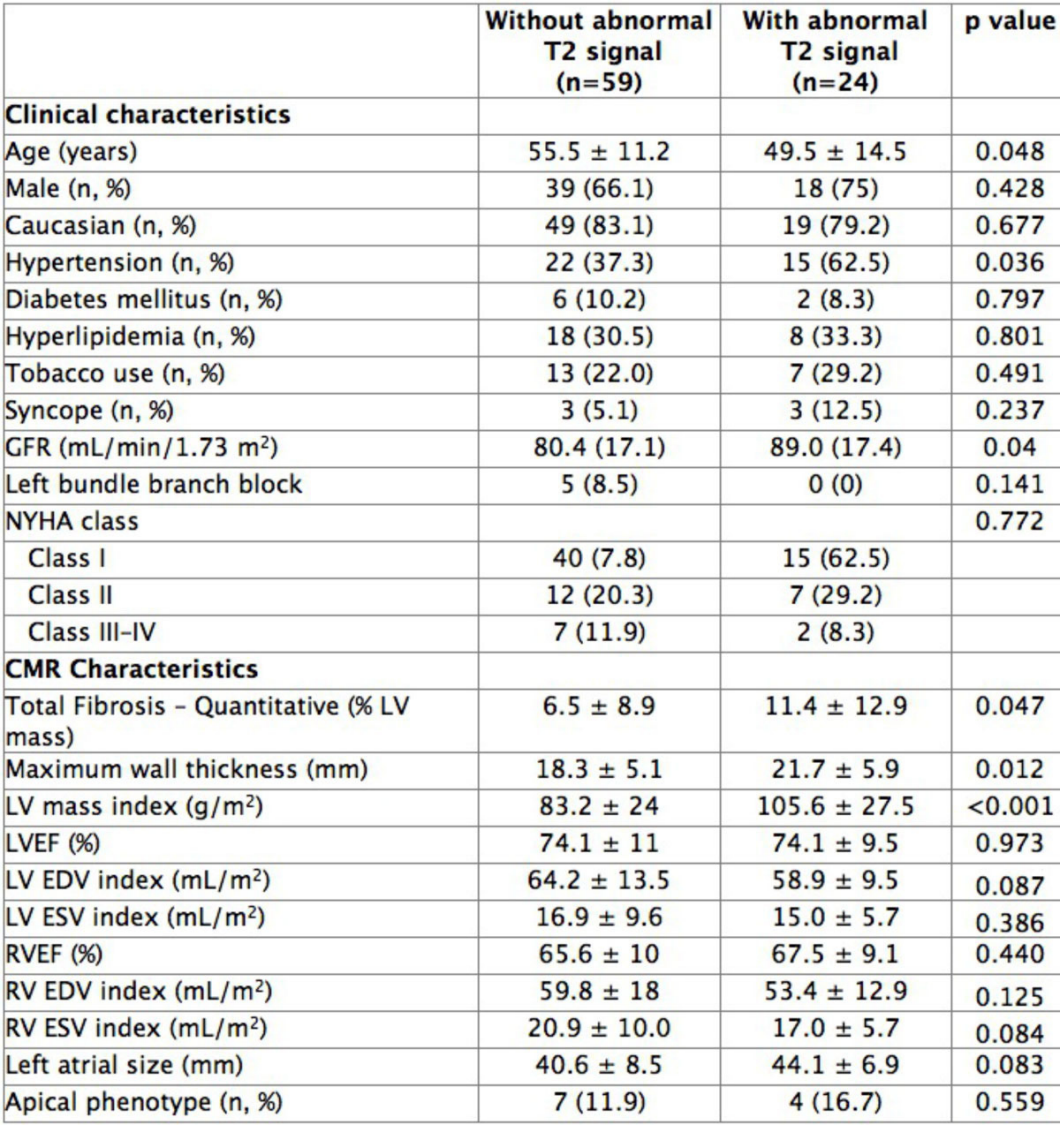
**Clinical and MRI-based characteristics of hypertrophic cardiomyopathy patients with and without abnormal T2 signal**.

**Figure 2:**
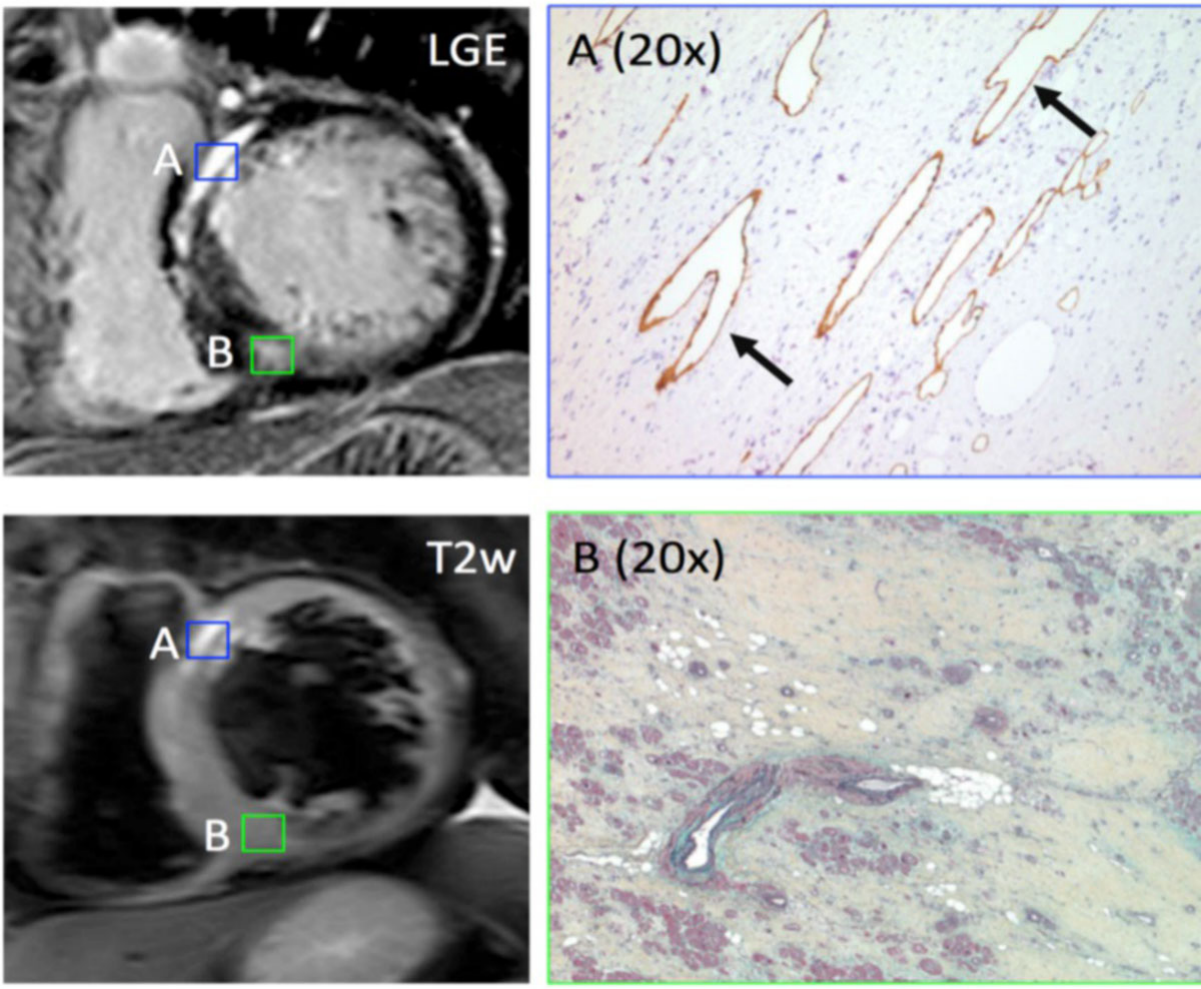
**Correlation of late gadolinium enhancement and T2-weighted (T2) imaging findings and histopathology in a 52 yo male undergoing cardiac transplantation**. Region of increased T2 signal [A] demonstrates large vascular channels embedded within dense fibrosis (arrows), staining positive for lymphatic endothelium (right panel). A reference region of fibrosis without abnormal T2 signal [B] demonstrates only fibrosis with scant fat deposits.

A total of 83 patients were studied (mean age 54.4 ± 12.5). T2 signal abnormalities were identified in 24 patients (29%). As shown in Figure 1, these patients were younger, had greater myocardial mass and maximal wall thickness, and a greater volume of myocardial fibrosis by LGE imaging. When present, abnormal T2 signal was reliably embedded within regions of dense fibrosis. Ex-vivo imaging confirmed high T2 signal and was used to guide histologic examination, showing the presence of unusual, large vascular channels. These were confirmed by immuno-histochemical staining to be lymphatic in origin (Figure 2).

## Conclusions

In the largest series to date of T2-weighted imaging in HCM, we identify that T2 signal abnormalities occur in one-third of patients and are associated with an advanced disease phenotype. These patients are typically younger with more myocardial hypertrophy and fibrosis. In a patient undergoing transplantation, T2 signal was histologically correlated to abnormal lympathic channels embedded in dense fibrosis. The capacity of these channels to contribute to re-entry mechanisms of arrhythmia in patients with HCM is of particular interest.

## Funding

None.

